# Accessibility Assessment of Community Care Resources Using Maximum-Equity Optimization of Supply Capacity Allocation

**DOI:** 10.3390/ijerph18031153

**Published:** 2021-01-28

**Authors:** Ming-Hseng Tseng, Hui-Ching Wu

**Affiliations:** 1 Department of Medical Informatics, Chung Shan Medical University, Taichung 40201, Taiwan; mht@csmu.edu.tw; 2 Department of Medical Sociology and Social Work, Chung Shan Medical University, Taichung 40201, Taiwan; 3 Social Service Section, Chung Shan Medical University Hospital, Taichung 40201, Taiwan

**Keywords:** community-based care access, accessibility, maximum equity, optimization, health resources, aging in place, healthcare services

## Abstract

Equity in accessible healthcare is crucial for measuring health equity in community care policy. The most important objective of such a policy in Taiwan is empowering people and communities by improving health literacy and increasing access to healthcare resources. Using the nearest-neighbor two-step floating catchment area method, this study performed an accessibility assessment for community care resources before and after supply capacity optimization. For the target of maximum equity when allocating community care resources, taking maximum values, mean values and minimum values of the distances into consideration, three analytical allocation solutions for supply capability optimization were derived to further compare disparities in geographical accessibility. Three indicators, namely, the Gini coefficient, median minus mean and mean-squared error, were employed to assess the degree of optimization of geographical accessibility scores at the locations of the demand population and to determine the degree of geographic inequities in the allocation of community care resources. Our study proposed a method in which the minimum value of the distance is adopted as the approximate representation of distances between the service point and the locations of demand to determine the minimum value for supply capacity optimization. The study found that the method can effectively assess inequities in care resource allocation among urban and rural communities.

## 1. Introduction

### 1.1. Health Equity, Community-Based Care Resources, and Aging in Place

Health equity has recently become an important issue in public health, in part because of the recognition of the degree to which personal health is affected by social, economic, and environmental factors. Based on the World Health Organization’s action guideline [1], “health equity is defined as the absence of unfair and avoidable or remediable differences in health among population groups defined socially, economically, demographically or geographically.” Health equity is the absence of unfair and avoidable differences in health between subgroups of a population. Health equity and health equality do not mean the same thing. Equality is focused on giving everyone the same treatment, whereas equity involves giving people what they need to reach their best health. The disparities of the people represent the unequal distribution of the social determinants of health in society. Therefore, identifying health inequalities and their drivers is essential for achieving health equity [2].

The World Health Organization proposed a conceptual framework for implementing action on the social determinants of health (SDH), which can be more important than healthcare or lifestyle choices in influencing health. Equity in SDH is a complex and multifaceted field. Moreover, certain challenges should be overcome in implementing action to address health inequities using the SDH [1,3]. Studies on SDH have found that the differences between health conditions of individuals or groups depend, in part, on their various economic and social situations. This includes income and wealth distribution, level of education, and the possession of power. Thus, the SDH are shaped by public policy, and social welfare policies and city resource allocation at the national and community levels can affect equity in healthcare deeply [4,5].

In 2002, the World Health Organization proposed a policy framework for active aging that emphasized policies to promote active aging. If the elderly are provided with the best opportunities to pursue health, social participation, and safety, their quality of life can effectively be promoted [6]. If active aging is put into place, the elderly can be helped to achieve successful aging. Phelan et al. [7] found that older people considered successful aging to involve the integration of a range of health conditions that depend on physical, functional, psychological, and sociability factors. In addition to medical services, social activities, which increase mental flexibility, and network-supporting connections, which strengthen health, also promote quality of life among the elderly. Therefore, active aging and successful aging are related to health equity. Due to their physical limitations, geographical accessibility affects the ability of the elderly to take advantage of community care resources, and this is reflected in equity in the design of resource allocation policies.

Monitoring health inequalities by investigating the observed differences in health between population subgroups is crucial to achieving health equity [2]. Health inequality monitoring uses health data disaggregated by relevant inequality dimensions such as demographic, socioeconomic, and geographical factors. For this reason, our study used a method involving maximum-equity optimization to identify those who are left behind in community care resource allocation.

The degree of health equity and access to equitable healthcare can be determined by such factors as the proximity of healthcare service points, equitable access to healthcare facilities, or equity in obtaining healthcare results [8]. Equity of access to healthcare is crucial for measuring health equity in community care policy [9,10,11,12,13]. Strengthening of community-based support, enhancement of resource accessibility, and design of resource allocation policies that aim for maximum equity are tools to accomplish the ideal of aging in place [9,11,12,14,15].

### 1.2. Spatial Optimization of Community-Based Care Resources

The most important objective of the community care policy in Taiwan is empowering people and communities by improving health literacy and increasing access to healthcare resources. Establishing ubiquitous community care stations can enhance the social participation of the elderly with better than sub-health status. Community networks can improve the physical and psychological health of the elderly, while personal and public medical outlays can also be lowered. Under these circumstances, the evaluation of the geographic accessibility of community care stations can indicate problems in the equity of the allocation of resources and can become an important reference for policymakers.

Community-based care resources are broadly defined. Community care stations, daycare providers, long-term care institutions, and medical institutions that are located in communities and serve the people who live there can be understood as community care resources in a broad sense. A search of the literature using the keywords “community care,” “elderly care,” and “geographic accessibility” produces reports that center discussions on the geographic accessibility of healthcare resources, and the related analyses highlight the variables of distance, population demand, and number of medical resource suppliers [16,17,18,19,20,21,22,23,24,25], which are factors that influence the utilization of home and community-based services among recipients of long-term care in Taiwan [26] as well as the accessibility of institutional healthcare facilities for the elderly [27,28,29]. Research on care resources in non-medical communities focuses on the business model of community-based care institutions [30] and the types of services provided to people with disabilities and the elderly [31]. In addition, local case studies assess the integration of family support and community care [32] and evaluate and investigate the geographic accessibility of community care stations [33] and the demand and supply allocation of community-based elderly learning resources [34]. However, less work was conducted on the optimization of the spatial allocation of community resources.

Methods of spatial optimization are frequently used to improve the distribution and supply of medical service providers. Wang [35] compared the methods of healthcare resource allocation optimization and found that solutions to classic location–allocation problems lie in the optimal effectiveness of resource allocation. Here, optimization targets would include maximizing coverage (in the maximum covering location problem), minimizing the number of facilities (the location set covering problem), minimizing total distance and time (the p-median problem), minimizing maximum distance (the center model), and minimizing inequity in accessibility (the equity model). These optimization methods can help improve the allocation plans of facilities related to community healthcare resources. For example, Tao, Cheng, Dai and Rosenberg [28] sought to optimize the allocation of elder care facilities, using the current spatial distribution of the elderly population in Beijing, China and a model constructed to achieve maximum equity. Liu et al. [36] integrated the two-step floating catchment area (2SFCA) method and the potential model to assess a better search radius. The study demonstrated that 600 m is close to the real travel distance of the elderly in Xi’an, China. Using the calculus concepts of the three-step floating catchment area method, Wu et al. [37] considered distances, capacity of hospitals, and Google ratings in an integrated manner. The authors demonstrated that the generated scores are in better accordance with people’s decision-making behavior when determining which physical rehabilitation resources to use in the community.

In the study of accessibility in community care resources, factors to be examined include the population at demand, locations of service points, number of service points, and the degree of coordination of distance factors. This study produced an evaluation method for geographic accessibility and set a target for maximum equity, from which three analytical optimal solutions for resource capacity allocation were derived. Using these solutions, the relevant results were analyzed and compared to determine whether the optimization of community care resource allocation could diminish the phenomenon of regional inequality. In this study, the analysis of population at demand was based on populations aged 65 and above in villages on the main island of Taiwan. Regarding the supply points for resources, the analysis was based on the number of community care stations on the main island of Taiwan that were accessible to nearby elderly people in sub-health conditions. The villages investigated supplied the statistical stratification basis. Furthermore, the results for various villages in different counties/cities were compiled and analyzed to discuss the allocation of community care stations in different counties/cities and disparities in resource accessibility for the population at demand in the investigated villages.

This study examined current distributions of people at demand and community care stations, as well as the accessible rate of these stations for the population at demand. Means of increasing the accessibility of community care resources for elderly people were also examined, with maximum equity as the target, by assisting the beneficiaries to look for treatment at the nearest facilities and to reduce the traffic obstacles they may encounter.

This study explored the following issues:

1. The spatial distribution of the population at demand in relation to the numbers of community care resources in target villages;
2. Taking the target of maximum equity with maximum values, mean values, and minimum values of distances into consideration, four community-care-capacity allocation methods for resource capability optimization were examined to compare disparities in geographical accessibility of community care resources;
3. Follow-up improvements to policies based on the differences in densities of community care resources in counties/cities were suggested.

## 2. Materials and Methods

### 2.1. Data Collection: Study Area and Datasets

The geographic area covered by the analysis in this study includes 19 counties/cities, 349 townships, and 7681 villages. Information on community care stations was retrieved from the open data of the Social and Family Affairs Administration at the Ministry of Health and Welfare, which were disclosed in 2017 on the ministry’s website for community care stations services [38]. Information on the population aged 65 and above in villages was retrieved from the Social and Economic Database of the NGIS Social and Economic Information Service, Ministry of the Interior, released in March 2017 [39].

Transportation is an important factor that determines senior citizens’ access to community care resources. However, to examine differences in the convenience of transportation among counties/cities, we considered types of vehicle, shift frequencies, travel times, fare policies, and fare subsidy policies in counties/cities. Due to the scarcity or low credibility of the relevant data, it did not prove feasible to incorporate such information into the analysis of road network data. In its evaluation of the factors that affect geographic accessibility, the study drew from the research methods of Page et al. [40]. To reduce possible errors, road network data, which represent actual route distances provided in government open data, were adopted as the basis for the analysis of transportation-influencing factors instead of traditional map distances (computed as the linear distance between two points). For cartographic data, numerical maps were taken from the Ministry of Transportation and Communications [41]. ArcGIS, which incorporates geographic information systems, was used to calculate geographic accessibility with network analyses. Because the geographic accessibility analysis focused on the convenience of users’ mobility, if data for supply points located on the main island of Taiwan are mixed with those of outlying islands and assessed collectively, deviations in resource-accessibility assessments can be expected. For this reason, the study area was limited to the main island of Taiwan.

The indicated data allowed the community care service points to be categorized into subsidized and functional points (this latter included general facilities, nursing homes, and home care facilities). Subsidized points generally receive subsidies to support their service delivery. Among the functional points, in addition to service points for the use of healthy elderly people and elderly people with sub-health conditions, service points providing nursing home and home care services were included. In this study, the screening range for the list of service points was limited to general functional points that receive subsidies. Research data up to November 25 were included in the statistics, and 2023 community care stations were located in the country, of which 1966 were located on the main island of Taiwan. Position data were confirmed. After service points with missing information or duplicated addresses were excluded, a final list of 1849 positions was produced, which provide service resources at 1854 points.

According to the Establish Community Care Station Implementation Plan of Taiwan [42], community care stations are called upon to provide at least three types of non-medical services, including home visits, phone calls, meal services, and health improvement activities. Thanks to the care they receive from the local community, seniors can engage more closely with society and come to live in familiar environments. The target group of services nearly encompassed all people aged 65 or above, including the healthy ones, the ones with a sub-health status, and the disabled elderly who need home care. Following the research objective for aging in place, in the estimation of the population at demand for community care stations in this study, the data analyses were refined down to 7681 villages on the main island of Taiwan as the basis for statistical stratification. Next, the results for various villages that belong to different counties/cities were compiled and analyzed to establish the allocation of community care stations in different counties/cities and disparities in the nearest resource accessibility of the population at demand in the corresponding villages.

### 2.2. Accessibility Calculation Using Analytical Solutions for Optimization of Supply Capacity Allocation

The geographic accessibility of resources forms a critical basis for the evaluation of resource allocation. Apparicio et al. [43] identified five commonly used measures of spatial accessibility, namely, distance to nearest service, number of services within a certain distance or time, mean distance to all services, mean distance to a certain number of nearby services, and the gravity model. The main limitation of the distance to the nearest service method is that it only captures proximity between population and service locations, without considering availability [44]. An important method for analyzing resource accessibility is calculating the ratio of resource allocation (number and locations) to the population at demand. Luo and Wang [45] proposed the 2SFCA method, which avoids the limitations caused by setting administrative regions as activity areas. This research method considers the potential for cross-region healthcare utilization as well as setting a reasonable range for seeking medical treatment, enabling an assessment of the spatial accessibility of medical resources. The 2SFCA method is primarily divided into two stages [23,44,46]. In stage one, the service loads for each service provider are calculated. In stage two, the resource-accessibility rates at each location of the population at demand are calculated to assess the geographic accessibility scores for the resources [20].

The 2SFCA method has its limitations. First, the method assumes that all services within the same catchment area are equally accessible to all people, which may not always be true because the attractiveness of a provider is dependent on number and service quality. Second, previous studies that utilized the 2SFCA method considered that all people with a catchment area use services equally, regardless of the characteristics of the population. As such, considering variations, such as age, socio-economic characteristics and the needs of seniors, is important when determining community care allocation [47,48,49].

The use of social welfare resources typically implies that the user has searched and selected a service provider within the available choices designated by policies and regulations due to limited government finances and resources. The present policy environment of Taiwan stipulates that the resources provided by community care stations within a county/city can only be used by the residents of that county/city, and each user can only visit one particular service point for a given service. Therefore, the authors of this study combined the domain partition OD cost matrix calculation approach with the nearest-neighbor 2SFCA (NN2SFCA) method in the calculations [33]. Adopting analytical solutions to optimize the supply capacity allocation as determined using the NN2SFCA, the current study assessed the optimization of accessibility to community care resources in favor of maximum equity, given the limitation of total capacities.

Using the NN2SFCA method, this study developed analytical solutions for the optimization of supply capacity allocation to calculate the geographical accessibility score as follows (for details of the derivation, please see Appendix A)

(1) $$A_{i}\;=\;\frac{S_{j}^{opt}*f\left(d_{ij_{NN}}\right)}{\sum_{k}P_{k}*f\left(d_{j_{NN}k}\right)}\;=\;\frac{f\left(d_{ij_{NN}}\right)}{R_{j}};\;R_{j}\;=\;\frac{\sum_{k}P_{k}*f\left(d_{j_{NN}k}\right)}{S_{j}^{opt}}$$

where *A_i_* is the geographical accessibility score for village *i* and implies the average amount of supply point resources enjoyed by each person at demand in the location of that village at demand. *R_j_* represents the service load for point *j*. $S_{j}^{opt}$ represents the optimal supply capacity for the point *j*. *P_k_* represents the size of the elderly population in the village’s location at demand *k*. $d_{ij_{NN}}$ is the route distance between the village’s location at demand *i* and the nearest-neighbor specific service point *j_NN_*. $d_{j_{NN}k}$ is the route distance between the nearest-neighbor specific service point *j_NN_* and the village’s location at demand *k*. *j_NN_* is the specific service point *j* found for the village’s location at demand *i* in a nearest-neighbor search. *f*(*d_ij_*) is the distance decay function, and the search radius for resources in this study is divided into two zones in relation to the respective distances. The first zone ($d_{ij}\leq \;3\;\mathrm{km}$) includes a range of points that an elderly person can reach by foot within 1 h [33]. The second zone ($d_{ij}>\;3\;\mathrm{km}$) includes the range of points that an elderly person can reach on foot in times over 1 h. *f*(*d_ij_*) is shown in Equation (2)

(2) $$f\left(d_{ij}\right)=\;\left\{\begin{array}{c} 1,\;d_{ij}=d_{ij_{NN}}\leq 3\;\mathrm{km}\\ \frac{3}{d_{ij}}\;d_{ij}=d_{ij_{NN}}>3\;\mathrm{km}\\ 0,\;d_{ij}\neq \;d_{ij_{NN}}\; \end{array}\right\}$$

In method M0, using the setting in which each supply point provides one unit of service capacity, the calculation assesses the disparity of spatial distribution in the supply of community care stations and the population at demand before the optimization of the supply capacity allocation.

(3) $$S_{j}^{opt}\;=\;1$$

In method M1, for each service point *j*, the maximum value for distance is adopted as the approximate representation of the distances between *j* and the locations at demand *i* that rely on its services, from which the maximum value for resource optimization capacity $S_{j}^{max}$ can be obtained, as shown in Equation (4)

(4) $$S_{j}^{max}\;=\;A_{e}*\underset{k}{\sum }P_{k}*f\left(d_{j_{NN}k}\right)*max_{i}\left(\frac{1}{f\left(d_{ij_{NN}}\right)}\right);\;A_{e}\;=\;\;\frac{S}{P}$$

where *A_e_*, the target of maximum equity, gives the ratio for the total supply capacity *S* to total demand population *P*.

In method M2, the average value for distance is adopted as the approximate representation of distances between *j* and the locations at demand *i* that relies on its services, from which the average value for resource optimization capacity $S_{j}^{avg}$ can be obtained, as shown in Equation (5)

(5) $$S_{j}^{avg}\;=\;A_{e}*\underset{k}{\sum }P_{k}*f\left(d_{j_{NN}k}\right)*avg_{i}\left(\frac{1}{f\left(d_{ij_{NN}}\right)}\right)$$

In method M3, the minimum value for distance is adopted as the approximate representation of the distances between *j* and the locations at demand *i,* which relies on its services, from which the minimum for of resource optimization capacity $S_{j}^{min}$ can be obtained, as shown in Equation (6)

(6) $$S_{j}^{min}\;=\;A_{e}*\underset{k}{\sum }P_{k}*f\left(d_{j_{NN}k}\right)*min_{i}\left(\frac{1}{f\left(d_{ij_{NN}}\right)}\right)$$

To meet the restriction of resources rendered by the original input total supply capacity *S*, resource optimization capacities go through standardized processing, as shown in Equation (7)

(7) $$S_{j}^{opt}\;=\;\frac{S}{\sum_{j}S_{j}^{k}}*S_{j}^{k},\;k\;=\;max,\;avg,\;min$$

Table 1 shows the geographical accessibility score evaluation equations used in methods M0–M3 of this study.

### 2.3. Inequality Indicators

In measuring inequality, the measures for mean, median and Gini coefficient are often applied [50]. The median is the middle number in a sorted list of numbers, with the same amount of numbers above and below it. The median is sometimes used in place of the mean in cases where there are outliers in the sequence that could skew the average. The median of a sequence is less affected by the values of outliers than the mean. The absolute value for median minus mean (|Median−Mean|) is the first indicator of inequality used in this study. Values of |Median−Mean| that are closer to 0.0 indicate smaller differences in the distribution of accessibility and better degrees of fairness.

The mean-squared error (MSE) measures the average squared difference between estimated and target values. It represents the disparity degree between the geographical accessibility scores in villages and the target value for maximum equity (average value for the entire island), where larger values represent larger disparities in resource distribution. MSE was also used to conduct a discrepancy evaluation for the geographical accessibility score optimization of the locations of the population at demand.

The Gini coefficient was defined by Italian statistician Corrado Gini using the Lorenz curve as a measure for the equality of income distribution within a society [51]. The Gini coefficient ranges from 1 to 0, where 1 represents complete inequality in annual income distribution, and 0 represents complete equality. Generally speaking, a Gini coefficient below 0.2 indicates a highly equitable income distribution, the range 0.2–0.3 is equitable, 0.3–0.4 is bearable, 0.4–0.6 represents serious inequality, and above 0.6 indicates high inequality [52]. For Gini coefficient values above 0.6, the administrative authority is advised to be on the alert for excessive income inequality, as this situation may lead to social conflicts. Due to this, the Gini coefficient is also termed the inequality coefficient.

The distribution of community care resources is unequal in Taiwan, especially between urban and rural districts. This study, therefore, used three inequality measures to compare accessibility scores among four community-care capacity allocation models. In summary, lower values of the three inequality indicators indicate better equity.

## 3. Results

### 3.1. Distribution of People at Demand for Villages and Community Care Resources

Table 2 provides an overview of resources using the regional average method. For the over-65 population at demand in counties/cities, the accessible rates of service points ranged between 0.096 and 1.626, and the accessible rate per thousand elderly of the entire island was 0.589. The accessible rates of service points in the villages per thousand elderly ranged between 8.91% and 45.49%, while the average value of the whole island was 24.14%. The six most urbanized municipalities (Taipei City, Kaohsiung City, New Taipei City, Taichung City, Tainan City, and Taoyuan City) hosted 66.69% of those aged 65 years and above, and the accessible rates of eight counties/cities were lower than the average value for the whole island. It is worth noting that in the three municipalities (Taipei City, Kaohsiung City, and New Taipei City) with the highest degree of urbanization and the highest density of the elderly population, the accessibility rates were all lower than the average value for the whole island. These data indicate that equity in the accessibility rates of community care stations for the elderly populations in highly urbanized counties/cities leaves much to be desired.

### 3.2. Optimization of Supply Capacity Allocation and Service Load Equality

Table 3 lists the allocation results for supply capacity (Sj) in 19 counties/cities before and after optimization (methods M0–M3). Please note that both before (method M0) or after (methods M1–M3) optimization, the total supply capacity is 1854 units. In Table 3, the value of the change is equal to the optimized capacity minus the capacity before optimization. The symbol “*” indicates a county/city with a change value greater than 0.0, which means that the supply capacity for the county/city after optimization must be increased. The number of counties/cities that need to increase the supply capacity was found to be eight for method M1, seven for method M2, and only six for method M3. On the other hand, the number of counties/cities that need to reduce supply capacity was 11 for method M1, 12 for method M2, and 13 for method M3.

Table 4 presents the service load (*R_j_*) estimations for every service point for the different optimization models. The resource service load for each service point before and after optimization indicate quite different resource-service loads at each point before optimization. After optimization, the resources service load for each service point may become more similar. A comparison of standard deviations indicate that the values decrease progressively from method M0 to M3, implying that method M3 can lower the disparity in the number of people that constitutes the service load. The results of method M3 for the comparison of the values for |Median−Mean| indicated only 22 people and also rendered the lowest value. As shown in Table 4, method M3, which estimates according to a minimum value of supply capacity optimization, can achieve the most equal service load across the whole community care stations under limited resources.

### 3.3. Assessment of the Degree of Inequality in Community Care Resource Distribution

The geographical accessibility assessment could provide a fair distribution in a policy allocating community care resources [16,17,18,20,22,23,24,33,35,44,45,53]. This study proposed three analytical optimal solutions for the optimization of supply capacity allocation and applied measures of inequality to assess the geographical accessibility of community care resources. In Table 5, geographical accessibility scores were calculated using four methods before optimization (method M0) and after optimization (methods M1–M3) to assess the degree of inequality present in the distribution of community care resources.

A comparison of the first six statistics listed in Table 3 indicate that the scores generated by method M3 have the highest median and the lowest standard deviation and range. This indicates that if the resource allocation was carried out according to method M3 (after optimization), the distribution of geographic accessibility scores would have a more uniform trend than that obtained through method M0 (before optimization).

The last three inequality indicators listed in Table 5 show that the three analytic solutions (methods M1–M3) are fairer than those before optimization (method M0). For example, the |Median−Mean| indicator is 0.365 before optimization (method M0), and after optimization, for method M1 it is 0.212, for method M2 it is 0.044, and for method M3 it is 0.050. In the indicator of the Gini coefficient, method M0 (before optimization) produced the highest value (0.670), and method M3 (after optimization) produced the lowest value (0.006). This indicates that if resource allocation before optimization is conducted according to method M0, the degree of inequality in the accessible rate of service point resources is the highest, while methods M1, M2, and M3 can improve equity in the accessibility of resources after the optimization of capacity allocation, among which M3 can reduce the degree of inequality to the greatest extent. The MSE value for method M0 was as high as 1.651, and in contrast, the value for method M3 was 0.015. This indicates that a resource allocation method that follows method M3 will minimize the accessibility disparities in community care resources available to populations at demand in different villages.

In Table 6, the quintile method is used to compare geographical accessibility scores obtained through the four assessment methods, revealing improvements in resource allocation equity. Within the Q5 and Q95 class intervals, the dispersions of resource distribution disparity are presented. The distribution area for method M0 was the largest, and method M3 had the lowest disparity. For Q25, Q50, and Q75, method M3 rendered the same value of 0.617. This indicates that if resource allocation assessment is carried out according to method M3, over 75% of the population at demand will have consistent accessibility and achieve the maximum equity target.

Using the quintile method, the geographical accessibility scores found in methods M0–M3 were divided according to a 20% class interval, and the spatial distributions of the accessibility scores of community care points were set using maps. From the lowest accessibility scores to the highest, the maps were marked with the colors red (0–20%), orange (21–40%), green (41–60%), light blue (61–80%), and deep blue (81–100%).

**Figure 1:**
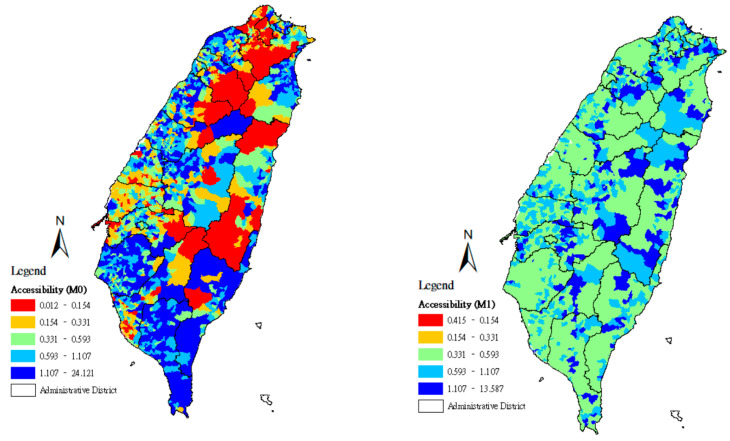
Accessibility score of community care resources in Taiwan using methods M0 and M1.

Figure 1 displays the map results for methods M0 and M1, where the lower-accessibility areas are scattered across eastern Taiwan and the mountainous zones of central Taiwan. The calculation of method M0 evaluates how the populations at demand in each county/city of Taiwan reach community care stations along the shortest routes, especially when the service points are distributed across metropolitan areas and districts with convenient transportation. In this case, the service point accessibility score of the population at demand in that county/city is higher. Figure 1 also shows that in the assessment by method M0, the inequality in resource allocation between counties/cities is considerably high. Lower-accessibility areas are mainly scattered across the two northern municipalities (Taipei City and New Taipei City). In method M1, for each service point *j*, the maximum value for distance is adopted as an approximate representation of the distances between *j* and the locations at demand *i*, which rely on its services, allowing the maximum value of resource optimization capacity $S_{j}^{max}$ to be derived. Here, Figure 1 exhibits the map result for method M1. That is, lower-accessibility areas disappear, and the most widely distributed areas are the moderate-accessibility areas, with high-accessibility areas only appearing in certain areas. Although the red and yellow areas determined by method M1 are significantly smaller than those of method M0, the three inequality indicators remain relatively large (Table 5).

**Figure 2:**
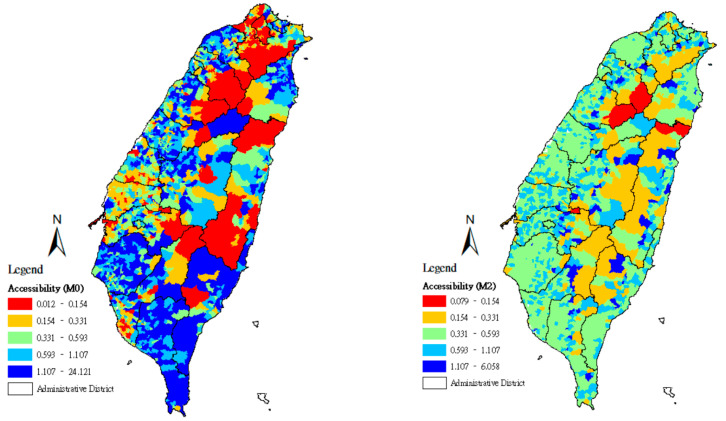
Accessibility score of community care stations in Taiwan using methods M0 and M2.

Figure 2 was mapped according to methods M0 and M2. For the results of method M2, the average value for distance is adopted as the approximate representation of the distances between *j* and locations at demand *i* that rely on its services, from which the average value of resource optimization capacity $S_{j}^{avg}$ can be derived. Accordingly, Figure 2 exhibits the map results for method M2. Moderate-accessibility areas are widely distributed, and lower-accessibility and higher-accessibility areas are presented only in some areas. A comparison of Figure 1 and Figure 2 indicates that when the maximum value for resource optimization capacity is adopted, the accessibility scores tend to reach the maximum value; when the average value of resource optimization capacity is adopted in the estimation, Figure 2 shows more moderate-accessibility areas than Figure 1; at the same time, areas of lower accessibility increase, which represents the way that choice of distance produces a considerable effect on the equity of resource accessibility.

**Figure 3:**
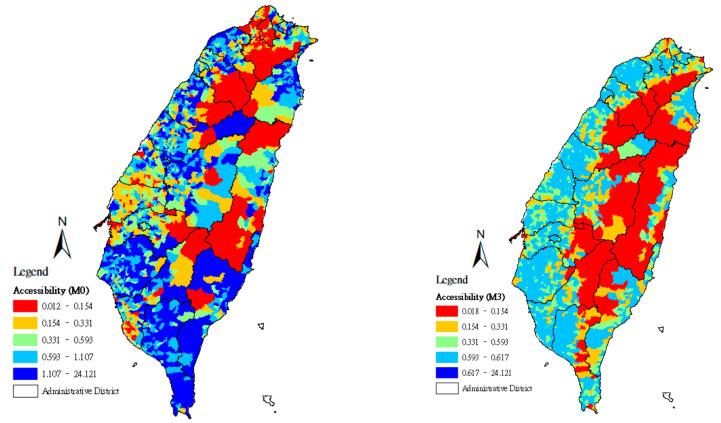
Accessibility score of community care stations in Taiwan using methods M0 and M3.

In Figure 3, minimum-distance values are adopted as the approximate representation of the distances between *j* and locations at demand *i* that rely on its services, from which the minimum value for resource optimization capacity $S_{j}^{min}$ can be derived. When the maximum equity for resource allocation is pursued with the minimum value for resource optimization capacity, due to population densities and the convenient transportation system in northern, western, and southern Taiwan, lower, moderate, and higher accessibility become scattered across a larger zone and reveal the possibility of maximum equity. In eastern Taiwan and the mountain zones, fewer community care stations appear, and all service points are located more than 3 km from villages with the population at demand. Thus, when the distance needed to reach a service point is represented by the location of the population at demand in a village through the shortest distance from the point, other locations of the population at demand at a distance value larger than the shortest distance are indicated as having lower accessibility. Figure 3 also shows that the government should prioritize improving problems in eastern Taiwan and the mountain zones, which have insufficient service points allocated to them, to promote the welfare of the elderly population in counties/cities with lower accessibility.

Using the absolute values for geographical accessibility scores rendered with |Median−Mean|, MSE, and the Gini coefficient, the differences in the resource allocation optimization models evaluated by the four methods are compared in Table 5 to indicate the degree of improvement in the maximum equity of community care accessibility in counties/cities. When |Median−Mean| is larger than the average value for |Median − Mean| for the whole island, the resource allocation of the county/city is uneven and therefore needs improvement. In method M0, four counties/cities had |Median−Mean| values larger than the average value for median value minus mean value over the entire island. The optimization models for methods M2 and M3 decreased |Median−Mean|. That is to say, the distributions of geographical accessibility in all counties/cities can be redistributed more even using the community-care capacity allocation optimization models M2 and M3.

Table 7 demonstrates that the MSE values for four counties/cities are greater than those for the average of the entire island by method M0, that is, these counties/cities’ accessibility scores are farther away from the fair target value (Ae). Moreover, the MSE values of the five counties/cities have increased in method M1, while methods M2 and M3 bring the accessibility distribution closer to the fair target value in all counties/cities after supply capacity is optimized.

The Gini coefficient was employed to compare the degree of improvement rendered by methods M0–M3 in resource allocation inequity among counties/cities. In all 19 counties/cities, Table 7 shows that M1–M3 make the Gini coefficient smaller due to the optimization of the supply capacity, indicating that geographical accessibility can become more equal. In the assessment of method M0, two counties/cities are found to have high inequity, and 17 counties/cities have median inequality. In methods M2 and M3, no counties/cities have inequitable distribution. In method M3, the value rendered from the Gini coefficient was the lowest among all optimization models, showing that method M3 is the best way to achieve maximum equity in resource allocation.

## 4. Discussion

The 2SFCA, proposed by Luo and Wang [45], breaks the limitations caused by setting administrative regions as activity areas. The research method considers the possibilities of cross-region healthcare utilization, and it also sets a reasonable range for seeking medical treatment, enabling assessment of the spatial accessibility of medical resources. The utilization of social welfare resources usually carries the implication that users must search and choose a service provider within a range of choices designated by policies and regulations. Present-day policy in Taiwan stipulates that the resources provided by community care stations in a county/city can only be used by the residents of that county/city, such that each user can only visit their nearest service point. For this reason, the current study compares approaches [33] (in the form of method M0) and considers the number of supplier resources (M1–M3) in relation to the NN2SFCA method. This study derives analytical solutions for the optimization of supply capacity allocation to minimize the discrepancies in the geographical accessibility scores for the locations of the population at demand to reach community care stations.

Previous publications have reported related studies. For instance, Tan et al. [28] used the numerical iteration method and Particle Swarm Optimization to optimize the number of beds at a residential care facility. This study applied mathematical methods and produced analytical solutions to optimize the supply capacity allocation for community care resources. Numerical solutions are slower trial-and-error and iteration procedures that result in approximate solutions. By contrast, analytical solutions are logical and direct procedures that yield a solution in an exact form. Therefore, the three optimization methods (methods M1–M3) used in this study are relatively concise and rapid for the accessibility optimization analysis.

Neighborhood social networks are important for enhancing the health and well-being of seniors, whereas community-based care is closely associated with their participation in social activities. In these social interactions, geographic accessibility is a significant factor. The 2SFCA method, which is based on an improvement in the early floating catchment area model, is significant for assessing the geographic accessibility of public facilities. Wu and Tseng [33] adopted the NN2SFCA method and further proposed a new method (i.e., calculated accessibility of the nearest distance-decay, which accounts for population of villages, supplier loading, and elderly walkability) to measure disparities in community care resources among cities/counties. However, the study overlooks the optimization solution to confront the inequality in geographic accessibility. In the same manner, Liu, Wang, Zhou and Kang [36] used the 2SFCA method and set a threshold (i.e., acceptable maximum for travel time or distance) to calculate the spatial accessibilities of community care facilities. The model was named the “potential model,” which pays more attention to the distance attenuation effect of spatial distance in terms of accessibility but overlooks inequalities in the capacities of community care resources.

Given government fiscal restraint and the increasing aged population, the provision of social welfare resources may be reduced. Thus, the current study proposed analytical solutions to optimize supply capacity allocation using NN2SFCA. The method can aid policy planners in assessing the optimization of the accessibility of community care resources under the limitation of total capacities.

The three optimization methods derived in this study can be compared as follows. (1) For resource-accessibility assessment of demand, in the order of pros and cons, M3 was the best, M2 was second, and method M1 was the worst, as determined by three inequality indicators of |Median−Mean|, MSE, and Gini coefficient. (2) For supplier load consistency evaluation, the ranking remains the same, based on the two consistency indicators of |Median−Mean| and MSE. In summary, this study indicated that method M3 is the best model to use for optimizing supply capacity allocation.

The study only incorporated spatial factors. However, other factors that influence the utilization of community care resources among seniors should be considered, such as social and economic conditions. In the future, research can weigh and add these multifaceted factors to calculations when analyzing well-rounded accessibility to determine and enhance the health equity of the elderly within the community care policy.

## 5. Conclusions

The degree of health equity and access to equitable healthcare can be determined by such factors as the proximity of healthcare service points, access to healthcare facilities, and ease in obtaining healthcare results. Health equity is the absence of unfair and avoidable differences in the status of health between subgroups of a population. Monitoring health inequalities by investigating the observed differences in health between population subgroups is crucial to achieving health equity. Active aging and successful aging are related to health equity. Because of seniors’ physical limitations, geographical accessibility affects the ability of elderly people to take advantage of community care resources, and this is reflected in equity in the design of resource allocation policies. Equity of access to healthcare is crucial for measuring health equity in community care policy. For this reason, our study used the method of maximum-equity optimization to identify the cities/counties where community care resource allocation needs to improve.

Adopting analytical solutions for the optimization of supply capacity allocation determined with the NN2SFCA, this study assessed how community care resource accessibility could be optimized in favor of maximum equity under a total capacity limitation. For strengthening community-based support, increasing resource accessibilities, and achieving the ideal of aging in place, this study makes contributions to policy implementation. Using method M3 (where the minimum value of distance is adopted as the approximate representation of distances between *j* and locations at demand *i* that rely on its services to find the minimum value for resource optimization capacity) proposed by this study, urban–rural disparities could be effectively lowered. The results of this study show that when the location of each service point is fixed and under the same amount of input resources, method M3 brings the population distribution of each demand point to the best geographical accessibility. Thus, M3 can help the government to effectively use the same amount of care resources to achieve maximum equity.

To improve healthcare practices and policy, this research suggests the following. (1) First, collect demand population, supply resources, and road network map, carefully checking the accuracy of the data. (2) Then, analyze the geographical accessibility of the current situation with method M1. (3) Assess the geographic accessibility after capacity allocation optimization using method M3. (4) Using the results of step 2 and step 3, evaluate the improvement of the first stage without adding more resources by comparing the three inequality indicators. (5) According to the results of the geographic accessibility analysis in step 3, determine the area with larger values of three inequality indicators and increase more new supply centers at appropriate locations within the area, which can be understood as the second stage of improvement.

Taking Taiwan′s current community care resources as an example, the policy recommendations of this study are as follows: Kaohsiung City currently has 137 community care stations. If the Kaohsiung City government provides 137,000 h/month, the central government can supplement this to 229,880 h/month (see Table 3). Then, the current Gini value of Kaohsiung City can effectively be reduced from 0.653 (high inequality) to 0.014 (highly equitable) (Table 7). After the first stage of improvement attained through applying M3, however, the three indicators of inequality at Hsinchu County and Hualien County still present relatively large values. Thus, a second stage of improvement should target Hsinchu County and Hualien County, and the central government should provide more funding to establish new community care stations in Hsinchu County and Hualien County (refer to Table 7).

Due to the restrictions to access to data and a lack of details, the limitations encountered in this study include the following. (1) The people at demand were positioned at the geometrically weighted center-points of the population. This can only provide reference locations and cannot reflect the exact locations of people at demand. The authors suggest that finer space scales be used, such as the basic statistical areas strata to improve investigation in the future. (2) Activity areas in geographical accessibility are represented only by route distances, and the values might not reflect exact travel times. In later studies, different vehicles can be used in the calculations and assessments. (3) This study only incorporated spatial factors. The relevant social and economic conditions can be weighted and added to the calculations to bring about well-rounded accessibility analyses. (4) Government open data do no not show the number of users at service points. Therefore, in relation to the differences in people at demand and the actual number of users, cross-validation was not possible for this study.

## Figures and Tables

**Table 1:** Definition of geographical accessibility calculation models.

Method	Description	Equation
NN2SFCA	Geographical accessibility score	$A_{i\;}=\frac{S_{j}^{opt}*f\left(d_{ij_{NN}}\right)}{\sum_{k}P_{k}*f\left(d_{j_{NN}k}\right)}$ $f\left(d_{ij}\right)=\left\{\begin{array}{c} 1,\;d_{ij}=d_{ij_{NN}}\leq \;3\;\mathrm{km}\\ \frac{3}{d_{ij}}\;d_{ij}=d_{ij_{NN}}>3\;\mathrm{km}\\ 0,\;d_{ij}\;\neq \;d_{ij_{NN}}\; \end{array}\right\}$ $A_{e}=\frac{S}{P}$
M0	Before optimization	$S_{j}^{opt}$ = 1
M1	Optimal supply capacity allocation taking the maximum operator	$S_{j}^{max}=A_{e}*\underset{k}{\sum }P_{k}*f\left(d_{j_{NN}k}\right)*max_{i}\left(\frac{1}{f\left(d_{ij_{NN}}\right)}\right)$ $S_{j}^{opt}=\frac{S}{\sum_{j}S_{j}^{max}}*S_{j}^{max}$
M2	Optimal supply capacity allocation taking the average operator	$S_{j}^{avg}=A_{e}*\underset{k}{\sum }P_{k}*f\left(d_{j_{NN}k}\right)*avg_{i}\left(\frac{1}{f\left(d_{ij_{NN}}\right)}\right)$ $S_{j}^{opt}=\frac{S}{\sum_{j}S_{j}^{avg}}*S_{j}^{avg}$
M3	Optimal supply capacity allocation taking the minimum operator	$S_{j}^{min}=A_{e}*\underset{k}{\sum }P_{k}*f\left(d_{j_{NN}k}\right)*min_{i}\left(\frac{1}{f\left(d_{ij_{NN}}\right)}\right)$ $S_{j}^{opt}=\frac{S}{\sum_{j}S_{j}^{min}}*S_{j}^{min}$

**Table 2:** Summary statistics for population aged 65+ or above and community care stations, measured by administrative district.

Administrative Districts	Population Aged over 65 Years	Percentage of Population Aged over 65 Years	Number of Centers	Number of Villages	Centers-to-Population (‰)	Centers-to-Villages (%)
Yilan County	69,013	2.19	74	233	1.072	31.76
Hsinchu County	65,305	2.07	38	192	0.582	19.79 *
Miaoli County	84,034	2.67	82	274	0.976	29.93
Changhua County	185,907	5.91	111	589	0.597	18.85 *
Nantou County	81,566	2.59	85	262	1.042	32.44
Yunlin County	119,761	3.80	50	388	0.417	12.89 *
Chiayi County	93,296	2.96	55	357	0.590	15.41 *
Pingtung County	127,325	4.04	207	455	1.626	45.49
Taitung County	32,837	1.04	49	140	1.492	35.00
Hualien County	49,484	1.57	32	177	0.647	18.08 *
Keelung City	53,550	1.70	59	157	1.102	37.58
Hsinchu City	49,406	1.57	26	122	0.526	21.31 *
Chiayi City	37,128	1.18	22	84	0.593	26.19
Taipei City	428,648	13.62	41	456	0.096	8.99 *
Kaohsiung City	383,659	12.19	137	891	0.357	15.38 *
New Taipei City	483,602	15.36	92	1032	0.190	8.91 *
Taichung City	310,710	9.87	237	625	0.763	37.92
Tainan City	265,121	8.42	291	752	1.098	38.70
Taoyuan City	227,931	7.24	166	495	0.728	33.54
Total	3,148,283	100.00	1854	7681	0.589	24.14

* Lower than average.

**Table 3:** Measures of supply capacity allocation of community care resources by method (M0–M3).

Administrative Districts	M0	M1		M2		M3	
	Capacity	Capacity	Change	Capacity	Change	Capacity	Change
Yilan County	74	38.789	−35.211	40.600	−33.400	40.730	−33.270
Hsinchu County	38	75.689	37.689 *	46.675	8.675	33.771	−4.229
Miaoli County	82	84.852	2.852 *	55.988	−26.012	47.332	−34.668
Changhua County	111	102.540	−8.460	104.785	−6.215	109.141	−1.859
Nantou County	85	62.167	−22.833	55.365	−29.635	44.334	−40.666
Yunlin County	50	81.021	31.021 *	70.562	20.562 *	65.978	15.978 *
Chiayi County	55	79.612	24.612 *	61.888	6.888 *	48.431	−6.569
Pingtung County	207	60.829	−146.171	72.606	−134.394	76.371	−130.629
Taitung County	49	23.561	−25.439	20.916	−28.084	17.926	−31.074
Hualien County	32	65.182	33.182 *	41.909	9.909 *	24.483	−7.517
Keelung City	59	23.627	−35.373	30.044	−28.956	32.634	−26.366
Hsinchu City	26	22.122	−3.878	27.472	1.472 *	30.356	4.356 *
Chiayi City	22	15.659	−6.341	20.520	−1.480	22.872	0.872 *
Taipei City	41	252.895	211.895 *	244.969	203.969 *	258.076	217.076 *
Kaohsiung City	137	193.957	56.957 *	220.179	83.179 *	229.880	92.880 *
New Taipei City	92	303.407	211.407 *	286.791	194.791 *	284.260	192.260 *
Taichung City	237	143.370	−93.630	174.578	−62.422	188.274	−48.726
Tainan City	291	120.098	−170.902	149.097	−141.903	160.918	−130.082
Taoyuan City	166	104.623	−61.377	129.056	−36.944	138.233	−27.767
Sum	1854	1854		1854		1854	

Note: * Change larger than 0.0.

**Table 4:** Measures of service load of community care resources through methods M0–M3.

						Estimated by People/Unit	
Method	Mean	Median	SD	Min	Max	Max–Min	∣Median–Mean∣
M0	1621	906	2449	14	31,649	31,636	715
M1	2080	2405	594	74	2405	2331	324
M2	1666	1813	321	159	1813	1654	147
M3	1600	1621	126	208	1621	1414	22

**Table 5:** Measures of geographical inequality of community care resources through methods M0–M3 (national level).

Estimated by 1000 × Capacity/People									
Method	Mean	Median	SD	Min	Max	Max–Min	∣Median–Mean∣	MSE	Gini Coefficient
M0	0.811	0.446	1.265	0.013	24.121	24.108	0.365	1.651	0.670
M1	0.628	0.416	0.715	0.416	13.587	13.171	0.212	0.512	0.327
M2	0.595	0.552	0.257	0.079	6.058	5.978	0.044	0.066	0.119
M3	0.567	0.617	0.121	0.019	0.617	0.598	0.050	0.015	0.006

**Table 6:** Quintile accessibility of community care resources measured through methods M0–M3.

Estimated by 1000 × Capacity/People					
Method	Q5	Q25	Q50	Q75	Q95
M0	0.071	0.191	0.446	0.933	2.752
M1	0.416	0.416	0.416	0.589	1.318
M2	0.393	0.552	0.552	0.573	0.861
M3	0.257	0.617	0.617	0.617	0.617

**Table 7:** Measures of geographic inequity of community care resources by methods M0–M3 (county level).

Administrative District	Method M0						Method M1						Method M2			Method M3		
	∣Median-Mean∣		MSE		Gini Coefficient		∣Median-Mean∣		MSE		Gini Coefficient		∣Median-Mean∣	MSE	Gini Coefficient	∣Median-Mean∣	MSE	Gini Coefficient
Yilan County	0.327		1.598		0.411	◎	0.268		0.596		0.451	◎	0.063	0.095	0.212	0.062	0.022	0.046
Hsinchu County	0.289		0.533		0.554	◎	0.510	∆	2.491	∆	0.509	◎	0.133	0.169	0.240	0.136	0.043	0.102
Miaoli County	0.287		1.561		0.484	◎	0.565	∆	4.181	∆	0.507	◎	0.101	0.136	0.151	0.086	0.025	0.026
Changhua County	0.218		0.494		0.503	◎	0.142		0.048		0.207		0.013	0.007	0.083	0.032	0.007	0.039
Nantou County	0.390	*	1.554		0.478	◎	0.361		1.078		0.485	◎	0.136	0.237	0.253	0.133	0.044	0.035
Yunlin County	0.136		0.161		0.421	◎	0.101		0.199	∆	0.244		0.011	0.022	0.109	0.071	0.015	0.070
Chiayi County	0.257		0.995		0.517	◎	0.236		0.786		0.352		0.080	0.251	0.204	0.129	0.039	0.078
Pingtung County	0.869	*	7.229	*	0.585	◎	0.096		0.105		0.231		0.029	0.031	0.092	0.036	0.012	0.014
Taitung County	1.406	*	18.417	*	0.740	◎◎	0.299		0.431		0.309		0.078	0.073	0.163	0.098	0.027	0.042
Hualien County	0.300		0.919		0.589	◎	0.737	∆	3.955	∆	0.544	◎	0.269	0.595	0.327	0.186	0.076	0.098
Keelung City	0.349		2.142	*	0.479	◎	0.024		0.033		0.040		0.006	0.005	0.016	0.012	0.004	0.015
Hsinchu City	0.124		0.481		0.455	◎	0.026		0.027		0.034		0.002	0.002	0.023	0.004	0.001	0.018
Chiayi City	0.217		0.839		0.425	◎	0.005		0.029		0.022		0.000	0.002	0.026	0.002	0.001	0.026
Taipei City	0.020		0.255		0.410	◎	0.174	∆	0.084		0.230		0.015	0.007	0.041	0.020	0.006	0.010
Kaohsiung City	0.248		0.801		0.653	◎◎	0.114		0.202		0.234		0.026	0.046	0.073	0.031	0.010	0.014
New Taipei City	0.096		0.199		0.528	◎	0.254	∆	0.299	∆	0.344		0.045	0.042	0.118	0.052	0.016	0.008
Taichung City	0.192		0.585		0.446	◎	0.058		0.065		0.150		0.011	0.012	0.052	0.019	0.006	0.014
Tainan City	0.483	*	3.333	*	0.574	◎	0.045		0.057		0.124		0.010	0.009	0.045	0.018	0.005	0.014
Taoyuan City	0.249		0.520		0.473	◎	0.060		0.151		0.148		0.018	0.038	0.051	0.019	0.007	0.016
National Average	0.365		1.651		0.670	◎	0.212		0.512		0.327		0.044	0.066	0.119	0.050	0.015	0.006

Notes: Estimated by 1000 * capacity/people. Level of distribution inequality estimated by │Median-Mean│ and MSE. *: larger than national average, Level of distribution inequality estimated by Gini coefficient. ◎: 0.4~0.6, median inequality, ◎◎: >0.6, high inequality, Level of distribution inequality estimated by∣Median-Mean∣ and MSE of M1~M3. ∆: larger than M0.

## Appendix A

This study adopted the concepts of the 2SFCA method (*I*(*j*) = 1). Equation (A1) is as follows

(A1) $$A_{i}=\;\underset{j}{\sum }\frac{S_{j}*I\left(j\right)*f\left(d_{ij}\right)}{\sum_{k}P_{k}*I\left(j\right)*f\left(d_{jk}\right)}$$

Based on the NN2SFCA model, *I*(*j*), as shown in the following equation, *j_NN_* is the specific service point *j* where a village’s location at demand *i* is found in a nearest-neighbor search.

(A2) $$I\left(j\right)\;=\;\left\{\begin{array}{c} 1,\;j\;=\;j_{NN}\;for\;each\;i\\ 0,\;j\;\neq \;j_{NN}\;for\;each\;i \end{array}\right\}$$

To achieve maximum equity in the capacity allocation of community care resources, a resource allocation optimization model that assumes that *Ai* = *Ae* (the target of the maximum equity) at every location at demand for resources is used. By combining Equations (A1) and (A2), can be adapted into Equation (A3)

(A3) $$A_{i}\;=\;\frac{S_{j}*f\left(d_{ij_{NN}}\right)}{\sum_{k}P_{k}*f\left(d_{j_{NN}k}\right)}\;=\;A_{e}$$

where $d_{ij_{NN}}$ is the route distance between the village’s location at demand *i* and the nearest-neighbor specific service point *j_NN_*, from which the analytical solution for the optimal supply capacity allocation can be derived, as shown in Equation (A4)

(A4) $$S_{j}\;=A_{e}*\frac{\sum_{k}P_{k}*f\left(d_{j_{NN}k}\right)}{f\left(d_{ij_{NN}}\right)}$$

Generally speaking, the total number of locations at demand *i* is larger than the total number of service points *j*. Each location at demand *i* will only find one *j**_NN_* point of shortest distance in the search, while each service point *j* will correspond to more than one location at demand *i*. Therefore, Equation (A4) may produce many solutions at each service point *j*. Therefore, the authors developed four analytical solutions for the optimal supply capacity allocation, i.e., methods M1–M3 in this study, as shown in Table 1.
